# Ocular Fungal Infections

**DOI:** 10.3390/jof8101078

**Published:** 2022-10-13

**Authors:** Max Carlos Ramírez-Soto, Alexandro Bonifaz

**Affiliations:** 1 Centro de Investigación en Salud Pública, Facultad de Medicina Humana, Universidad San Martín de Porres, Lima 15102, Peru; 2 Facultad de Ciencias de la Salud, Universidad Tecnológica del Perú, Lima 15046, Peru; 3 Dermatology Service & Mycology Department, Hospital General de México, “Dr. Eduardo Liceaga”, Balmis 148, Colonia Doctores, Ciudad de México 06726, Mexico

## 1. Introduction

Fungal infections of the eye continue to be an important cause of ocular morbidity and loss of vision, particularly in the developing world [1]. These infections have increased in recent decades due to broad-spectrum antibiotic use, the growing number of patients undergoing procedures that lead to immunosuppression, postoperative infection, trauma, and prolonged corticosteroid use [2]. Ocular fungal infections are categorized by the anatomical location of the infection. These infections can occur around the eye (ocular adnexa), or in the eye, including the anterior and posterior segments of the eye [3].

Major pathogenic fungi of the eye include *Aspergillus*, *Candida* spp., *Cryptococcus* species, and *Coccidioides* spp., *Fusarium, Penicillium, Pseudallescheria*, dimorphic fungi as *Histoplasma capsulatum*, *Blastomyces dermatitidis*, *Sporothrix* spp., and *Coccidioides* spp. (*C. immitis* and *C. posadasii*) [3,4]. The diagnosis of ocular fungal infections can be difficult because of non-specific clinical manifestations. However, in recent years it has been improved by laboratory and diagnostic techniques, and the recognition of the clinical signs of ocular fungal infections [4]. This has increased the frequency of correct diagnosis and prevalence of these diseases. Because of this, it is important to maintain to knowledge of new developments in the diagnosis and management of infectious diseases of the eye. In this setting, in this Special Issue, articles have been published describing novel findings and reviews on the epidemiology, diagnosis, and treatment of ocular fungal infections, with a special focus on infections in ocular adnexa, endophthalmitis, keratitis, and ocular sporotrichosis.

## 2. Fungal Infections in the Ocular Adnexa

**Figure 1:**
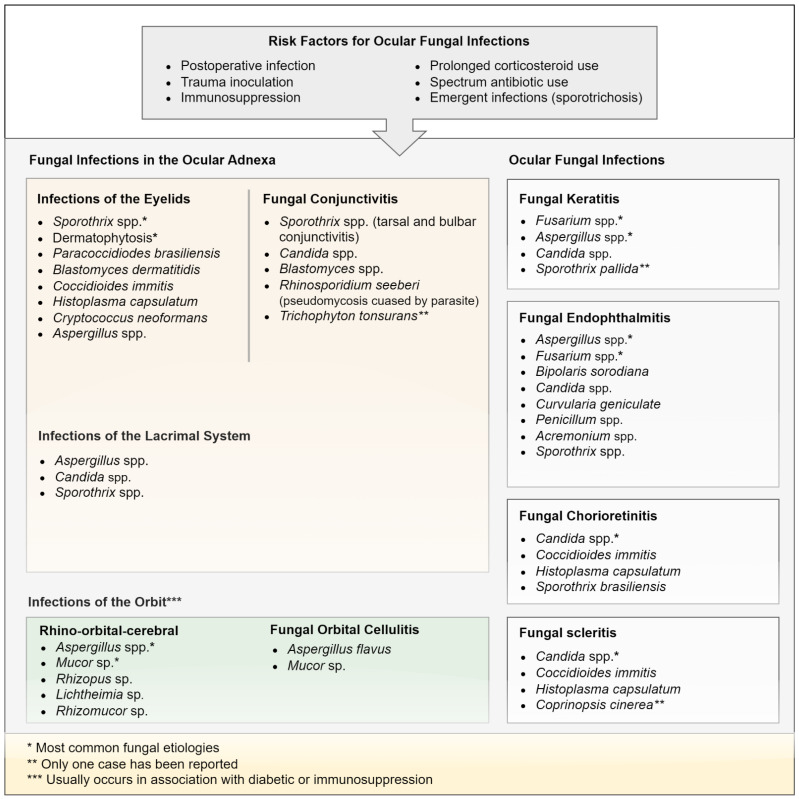
Causative aetiological agents from ocular fungal infections.

Fungal infections in the ocular adnexa include a wide variety of fungal diseases that involve the eyelid, conjunctiva, and lacrimal system, and may range from benign conditions to severe infections associated with systemic infections [3,4,5]. They are diagnosed based on the pattern involvement, clinical features or associated systemic findings, and epidemiological data. The most common pathogens vary by type of infection (Figure 1). Among fungal infections in the ocular adnexa, sporotrichosis (lymphocutaneous and fixed cutaneous), blepharitis and paracoccidioidomycosis seem to be the most common palpebral infections in the literature published [6,7,8,9]. Other palpebral fungal infections include: blastomycosis, coccidioidomycosis, histoplasmosis, cryptococcosis and aspergillosis [10,11,12,13,14]. Unlike fungal infections of the eyelid, fungal conjunctivitis is a rare disorder in ophthalmic care because of its low incidence and its unspecific clinical findings. *Candida* species are the major pathogens of fungal conjunctivitis [15,16], but it may also be caused by *Sporothrix* spp. [17] (Figure 1). Most cases are self-limited, but may also be associated with systemic infections. Mechanical trauma and immunosuppression may render the conjunctiva more susceptible to infectious agents.

Among fungal infections in the ocular adnexa, infection of the lacrimal sac and orbital fungal infection are uncommon causes of inflammation [16]. Infections of the lacrimal system include dacryoadenitis, canaliculitis and dacryocystitis. They are caused by primary endogenous infection or by organisms ascending to the lacrimal system [3]. Orbital fungal infections most commonly originate from adjacent paranasal sinuses, but they can result from other local or distant infection sources. They can be subdivided into preseptal infections and postseptal infections, usually called orbital cellulitis [3] (Figure 1). In this special issue of the journal, a review describes the epidemiological findings, clinical, diagnosis, and treatment of ocular sporotrichosis, a subcutaneous mycosis, which can occasionally result in an ocular condition [18]. The findings reveal that between infections in the ocular adnexa, palpebral sporotrichosis is the most common clinical manifestation followed by conjunctivitis and dacryocystitis. These infections usually occur in hyperendemic areas of sporotrichosis such as Brazil, China, and Peru. This study also aims to describe the most common findings of conjunctival sporotrichosis, and the differences between tarsal and bulbar conjunctivitis caused by *Sporothrix* [18].

## 3. Fungal Eye Infections

Fungal eye infections are uncommon and occur in different areas of the eye (Figure 1). Those located in the front layer of the eye are known as fungal keratitis (FK) and those that occur inside of the eye are known as fungal endophthalmitis [16]. Among ocular fungal infections, fungal FK is a chronic and indolent infection in hot, humid tropical climates. It accounts for 30–50% of all cases of microbial keratitis in developing countries. Risk factors include having sustained injury (by implantation) with plant material, use of corticosteroids, diabetes, etc. [19,20]. Clinical findings of FK may vary considerably, therefore, their diagnosis and management can be challenging. In this special issue of the journal, four articles provided information on FK. Because of the diversity of fungal aetiology, and the emergence of new corneal pathogenic fungi with varying drug susceptibilities, Raj et al. review the literature and discuss the most recent concepts in the diagnosis and management of FK, including microbiological and molecular diagnosis, antifungal susceptibility testing, and the outcomes of antimycotic therapeutic regimes, corneal collagen crosslinking, and penetrating keratoplasty [21]. Mayya et al., propose a multi-scale convolutional neural network for accurate segmentation of the corneal region to enable early FK diagnosis, and a ResNeXt model to differentiate between FK and non-FK. Their model on the segmented images in the corneal region achieved a diagnostic accuracy of 88.96% [22]. Chongkae et al., evaluated temporal trends and risk factors in the FK cases in a tertiary referral center in northern Thailand, as well as the changes in the spectrum of the aetiological agents, and virulence factors of FK [23]. Finally, Huang et al. performed molecular identification, and to assess the antifungal susceptibility of *Fusarium solani* species com-plex (FSSC) in the keratitis patients in Taiwan, and found that its minimal inhibitory concentrations for natamycin, voriconazole, chlorhexidine, lanoconazole, and luliconazole were higher in FSSC than those of non-FSSC [24].

On the other hand, fungal endophthalmitis is a devastating ocular inflammatory process inside the eye involving the vitreous and/or aqueous humors [25]. Unlike bacterial infection, fungi are an uncommon cause of endophthalmitis. Endophthalmitis can result from endogenous or exogenous sources. Most cases are exogenous and occur after ocular surgery, after globe injury or trauma, or after intravitreal injections. Endogenous endophthalmitis occurs after hematogenous dissemination of fungus [25]. In this special issue of the journal, two additional articles provided information on endophthalmitis. Haseeb et al., presented an updated review on the diagnosis and management of fungal endophthalmitis, whose prevalence is lower than bacterial endophthalmitis and clinically threatens patients’ vision [26]. Another review describes the differences in clinical and epidemiological characteristics and the differential diagnosis of exogenous and endogenous endophthalmitis caused by *Sporothrix* [18].

Finally, in this special issue of the journal, Sakamoto et al. described the clinical characteristics and risk factors in patients with ocular candidiasis vs. non-ocular candidiasis in a retrospective cohort study in Japan. Their findings reveal that *C. albicans* infection, an unremoved central venous catheter (CVC), and a high (1,3)-β-D-glucan value were associated with ocular candidiasis. In addition, unremoved CVC was detected as an independent risk factor for 30-day mortality [27].

## 4. Conclusions

In this special issue, reviews and articles have been published to summarize the latest research on fungal keratitis, endophthalmitis, ocular sporotrichosis and candidiasis. These studies and their clinical and epidemiological findings provide evidence to improve the diagnosis of ocular fungal infections and their management in ophthalmological practice. Finally, we wish to express thanks to all the authors and reviewers for their pivotal contributions to this special issue.
